# Improving the cryo-resistance of Egyptian buffalo semen via chlorogenic acid and rutin supplementation: molecular docking, antioxidant, and anti-apoptotic pathways

**DOI:** 10.1186/s12917-026-05896-9

**Published:** 2026-09-21

**Authors:** Wael A. Khalil, Aya A. Ismail, Mohamed M. Hegazy, Mostafa A. El-Harairy, Amany Assem Elkashef, Mahmoud Moussa, Sameh A. Abdelnour

**Affiliations:** 1 Department of Animal Production, Faculty of Agriculture, Mansoura University, Mansoura, 35516 Egypt; 2 Animal Production Research Institute, Agriculture Research Centre, Ministry of Agriculture, Dokki, Giza 12619 Egypt; 3 Chemistry Department, Faculty of Science, Alexandria University, Alexandria, Egypt; 4 Emirates Smart Camel Center, Umm Al Quwain, United Arab Emirates; 5 Department of Theriogenology, Faculty of Veterinary Medicine, Suez Canal University, Ismailia, Egypt; 6 Department of Animal Production, Faculty of Agriculture, Zagazig University, Zagazig, 44511 Egypt

**Keywords:** Buffalo semen, Antioxidant, Cryo-damages, Apoptosis, Molecular docking, Microbial load

## Abstract

This study compared the antioxidant effects of chlorogenic acid (CGA) and rutin (RU) supplementation in freezing extenders on the cryo-resistance of buffalo spermatozoa. Over eight weeks, 56 ejaculates were collected from seven fertile buffalo (*Bubalus bubalis*) bulls. To eliminate individual animal variability, high-quality ejaculates were pooled and extended in a Tris–egg yolk medium. The pooled semen was divided into three treatment groups: control (no additive), 0.6 mM RU, and 150 µM CGA. All groups were cryopreserved using a standard protocol and stored in liquid nitrogen for one month prior to post-thaw analysis. The findings revealed that the addition of either RU or CGA substantially enhanced post-thaw sperm viability, progressive motility, plasma membrane integrity, and acrosomal integrity compared with the control group (*P <* 0.05). Furthermore, both treatments improved sperm kinematics and antioxidant profiles by significantly increasing total antioxidant capacity and catalase levels (*P <* 0.05). This was accompanied by a significant decline in oxidative markers (MDA, ROS, and H_2_O_2_) (*P <* 0.05), indicating robust antioxidant activity. Supplementation with RU or CGA significantly increased sperm viability and notably reduced apoptotic percentages (*P <* 0.05). Furthermore, CGA treatment resulted in the lowest total bacterial and *coliform* counts among all experimental groups (*P <* 0.05). Molecular docking predictions suggest a potential interaction between CGA and RU against target proteins antioxidant status (HSP70) and mitochondrial function (mitochondrial uncoupling protein 1 and mitochondrial cytochrome c) in sperm. Overall, supplementing with rutin and chlorogenic acid offers robust protection against cryodamage. By mitigating oxidative stress and preserving membrane stability, these antioxidants substantially enhance post-thawed quality, and physiological function of buffalo spermatozoa.

## Introduction

The Egyptian buffalo (*Bubalus bubalis*) is a cornerstone of Egypt’s domestic livestock sector, playing a vital role in supplying high-value protein. Furthermore, this species is essential to the sustainable development of the country’s rural region [1]. Its physiological resilience and adaptability to local environments make it an indispensable asset to rural farming livelihoods [2]. Despite these advantages, the species faces significant reproductive challenges specifically silent estrus, prolonged postpartum intervals, and delayed puberty which collectively hinder optimal reproductive efficiency [3]. Applying assisted reproductive technologies (ARTs), such as sperm cryopreservation [4], in vitro oocyte maturation (IVM) [5], in vitro embryo production (IVEP) [6], and gene editing [7] can remarkably enhance reproductive efficiency and overcome these inherent biological limitations. Ultimately, integrating advanced sperm cryopreservation protocols is essential for optimizing reproductive performance and ensuring the genetic conservation of Egyptian buffalo populations [8]. Nevertheless, the cryopreservation process causes significant cellular damage, leading to irreversible ultrastructural defects and mitochondrial dysfunction that impair sperm fertility [9]. Spermatozoa experience multiple concurrent stressors, including thermal shock, ice crystal nucleation, osmotic shifts, and oxidative stress [10, 11]. Among these, oxidative stress (OS) is a primary culprit, destabilizing cellular membranes, damaging nuclear DNA and proteins, and severely disrupting mitochondrial architecture [8, 12]. Cryopreservation-induced stress triggers substantial alterations in mitochondrial ultrastructure [13], which compromises kinematic parameters and overall viability while promoting extensive DNA fragmentation [11, 14, 15]. Oxidative stress plays a central role in impairing sperm function by inducing morphological damage and degrading cellular DNA, membranes, and proteins [15]. Compounding this biochemical stress, physical and osmotic injuries from ice crystal nucleation and membrane destabilization independently threaten cellular viability [16], illustrating the multifactorial nature of cryoinjury [17]. This vulnerability is further exacerbated in buffalo spermatozoa due to the high polyunsaturated fatty acid (PUFA) content of their plasma membranes, which makes them highly susceptible to lipid peroxidation and cytotoxic byproduct formation [9, 18, 19]. Supplementing freezing extenders with exogenous antioxidants provides a targeted strategy to counteract these detrimental effects [20, 21]. Antioxidants neutralize ROS, thereby preventing lipid peroxidation and maintaining the morphological and functional integrity of post-thaw sperm [22]. In this context, flavonoids a broad class of polyphenolic plant compounds are frequently employed for their strong ROS-scavenging capacity [23, 24]. Notably, the antioxidant potency of flavonoids is heavily governed by their specific chemical structure, particularly the number and spatial orientation of their hydroxyl groups.

Rutin (RU), a flavonoid glycoside also known as rutoside or quercetin-3-O-rutinoside, is abundant in natural sources such as buckwheat, citrus fruits, and tea. Extensive research highlights its powerful ability to scavenge oxidative stress [25], inhibit apoptotic [26], and anti-inflammatory [27] properties. By directly donating electrons to neutralize free radicals [26], RU exerts a well-documented protective effect on male reproductive parameters [26, 28, 29]. Specifically, supplementing extenders with RU strengthens antioxidant defenses and shields boar sperm from oxidative damage during cryopreservation [30]. Similarly, Najafi et al. [31] demonstrated that RU supplementation significantly enhanced the post-thaw quality of ram epididymal spermatozoa. At the molecular level, RU exhibits potent anti-apoptotic effects by downregulating pro-apoptotic markers, such as Caspase-3 and Bax, while upregulating the anti-apoptotic protein Bcl-2 [27]. Chlorogenic acid (CGA) is a bioactive phenolic acid abundant in dietary staples such as coffee and tea. As a versatile polyphenol with potent antibacterial, antioxidant, and anti-inflammatory properties, CGA sees widespread application across the food, cosmetic, and pharmaceutical industries [32]. In reproductive biology, CGA supplementation improves progressive motility while reducing DNA damage and oxidative stress markers in preserved human semen [33], protective effects largely driven by its antioxidant activity, which is likely mediated through activation of the Nrf2 signaling pathway. Consistent with these previous studies, incorporating CGA into semen extenders significantly enhances sperm quality by bolstering antioxidant defenses and mitigating oxidative stress in both human [33] and ram semen [34]. Beyond male gametes, CGA significantly alleviates the detrimental impact of heat stress by modulating the testicular glutathione metabolism pathway [35]. Additionally, CGA supports oocyte development by upregulating intracellular thiol levels and enhancing glutathione peroxidase activity [36]. While Mohammadi et al. [12] established that supplementing extenders with RU and CGA improves post-thaw antioxidant status and in vivo fertility in buffaloes, critical mechanistic insights are still lacking. To date, no study has evaluated how these bioactive compounds influence sperm apoptotic cascades, acrosomal retention, intracellular ROS dynamics, or the extender microflora. Moreover, in silico molecular docking has not been leveraged to identify their specific target protein interactions. The present study addresses these unexplored frontiers to provide a comprehensive cellular and computational framework for antioxidant-mediated cryoprotection in Egyptian buffalo spermatozoa. Therefore, this study comprehensively aimed to compare the effects of rutin and chlorogenic acid supplementation on post-thaw sperm quality, kinematics, acrosome integrity, apoptotic status, antioxidant capacity, oxidative markers, and the microbial profile of cryopreserved buffalo semen. Furthermore, in silico molecular docking analyses were performed to evaluate the binding interactions of these compounds with key target proteins regulating spermatozoal mitochondrial bioenergetics and antioxidant pathways.

## Materials and methods

### Material sources and ethical approval

Chlorogenic acid (CGA) and rutin (RU) were purchased from Nawah Scientific Co. (Cairo, Egypt). All experimental procedures involving animals strictly complied with Directive 2010/63/EU of the European Parliament and of the Council (September 22, 2010) on the protection of animals used for scientific purposes. The study protocol adhered to the ARRIVE guidelines 2.0 and was formally approved by the Mansoura University Animal Care and Use Committee (Approval Code: MU-ACUC, AGR.RP.24.10.2). All methods were executed in accordance with institutional guidelines and regulations for animal welfare. Animals were sourced from Mahlat Moussa Station, Kafr El-Sheikh Governorate, belonging to Animal Production Research Institute, Agricultural Research Centre, Ministry of Agriculture, Dokki, Giza, Egypt.

### Buffaloes bulls and semen collection

Seven healthy, mature Egyptian buffalo bulls (*Bubalus bubalis*), aged 4–6 years, were used in this study. The bulls were housed at Mahlat Moussa Station, Kafr El-Sheikh Governorate, Egypt, under uniform management conditions and fed a balanced diet formulated to meet their maintenance requirements.

Ejaculates were collected once weekly using an artificial vagina maintained at 42–45 °C, yielding a total of 56 ejaculates (eight ejaculates per bull). Only ejaculates meeting strict quality criteria specifically, > 70% progressive motility, a sperm concentration of 8 × 10^8^ cells/mL, and < 15% morphological abnormalities were selected for further processing. Ejaculates from the seven bulls were pooled within each weekly collection session to generate eight independent biological replicates. Each weekly pool was then divided among the three experimental treatments. Selected ejaculates were pooled and diluted at 37 °C in a Tris-based extender to achieve a final concentration of 80 × 10^6^ spermatozoa/mL. Sperm progressive motility was independently evaluated by two experienced observers using a phase-contrast microscope equipped with a 100 × objective.

### Extender preparation, and freezing protocol

The semen freezing extender was prepared according to standard procedures at the International Livestock Management Training Center (ILMTC) and consistent with our previously described protocols [8, 18]. Briefly, the Tris-citric-egg yolk extender consisted of citric acid (1.675 g), penicillin (100 IU/mL), glycerol (6 mL), egg yolk (20 mL), Tris (3.028 g), fructose (1.25 g), and streptomycin (100 µg/mL) in double distilled water, with the final volume adjusted to 100 mL. Prior to the addition of cryoprotectants, the base diluent exhibited an osmolality of 280–300 mOsm/kg, measured using a micro-osmometer (Berlin, Germany), and a pH range of 6.7–6.9. Before semen extension, the extender was gently homogenized and pre-warmed to 37 °C in a water bath to achieve thermal equilibrium. The experiment was structured into three treatment groups as follows:

- The first treatment functioned as the control, utilizing a basal Tris-based extender without any supplementation.
- The second treatment consisted of a basal Tris-based extender fortified with 0.6 mM rutin (RU).
- The third treatment utilized a basal Tris-based extender fortified with 150 µM chlorogenic acid (CGA).

Supplementation doses for RU and CGA were chosen according to prior literature reporting peak efficacy for sperm quality parameters and in vivo fertility rates in buffaloes [12]. Semen samples were extended at a 1:10 ratio (v/v) to achieve a final concentration of 8 × 10^7^ spermatozoa/mL. Following dilution, the samples were gently homogenized and equilibrated at 5 °C for 4 h. The equilibrated semen was then loaded into 0.5 mL plastic straws (IMV Technologies, L’Aigle, France) and cryopreserved by exposure to liquid nitrogen vapor (placed 4 cm above the liquid nitrogen surface) for 10 min, before direct immersion into liquid nitrogen for long-term storage (4 weeks). Thawing was performed by immersing the straws in a 37 °C water bath for 30 s.

### Semen analysis

Sperm progressive motility (SPM) was assessed using a phase-contrast microscope equipped with a thermostatic stage maintained at 37 °C, following the methods previously described [18]. Sperm morphology and viability were determined utilizing the one-step eosin-nigrosin staining system [37]. Briefly, 10 µL aliquots of extended semen were mixed with the stain on a pre-warmed slide and examined under light microscopy at 400× magnification. At least 200 spermatozoa were counted per slide to determine viability: live cells remained unstained (white), whereas dead cells were stained red/pink due to membrane damage. Concurrently, head and tail abnormalities were evaluated to assess morphological integrity.

Sperm plasma membrane integrity was evaluated using the hypo-osmotic swelling test (HOST) at an osmolality of 75 mOsmol/L [19]. Briefly, a 50 µL aliquot of diluted semen was mixed with a 500 µL of HOST solution and incubated at 37 °C for 30 min. Following incubation, 10 µL drop of the suspension was examined under phase-contrast microscopy. Spermatozoa exhibiting characteristic tail swelling or coiling were identified as having functionally intact plasma membranes, consistent with previously established criteria.

### Motion parameters using CASA

Sperm kinematic parameters were evaluated using a Computer-Assisted Sperm Analysis (CASA) system (SpermVision, Minitube, Tiefenbach, Germany) according to the methodology described by [38]. Approximately 1,500 spermatozoa per treatment were evaluated at 37 °C. The system was coupled to an Olympus BX microscope (Hamburg, Germany) equipped with a rapid-scan digital camera and a 4 × dark-field objective to capture images at a frame rate of 60 Hz. The following sperm kinematic variables were assessed: distance straight line (DSL, µm), linearity (LIN = VSL/VCL), distance curved line (DCL, µm), velocity average path (VAP, µm/sec), distance average path (DAP, µm), straightness (STR = VSL/VAP), velocity straight line (VSL, µm/sec), velocity curved line (VCL, µm/sec), wobble (WOB = VAP/VCL), beat cross frequency (BCF, Hz), and amplitude of lateral head displacement (ALH, µm). To enable accurate automated assessment of kinematic characteristics, images were acquired at 60 Hz via a digital camera coupled with the microscope. For the CASA analysis, the frame rate was set to 25 fps, and the detection sensitivity was adjusted to a minimum sperm-head area of 15 μm².

### Acrosome status

Acrosomal integrity was assessed utilizing the Giemsa staining technique. Briefly, frozen-thawed semen samples were centrifuged and washed with albumin-free modified Oliphant and Brackett (mOB) medium to eliminate residual cryo-extender [39]. Following centrifugation and washing, thin smears were prepared on glass slides, air-dried, and incubated with a 10% Giemsa solution for 60 min. The slides were subsequently rinsed under distilled water, dried, and examined via bright-field microscopy at 1000 × magnification under oil immersion to assess acrosomal status.

- Intact/Viable: Sperm cells displaying pink-purple acrosomes and clear, white post-acrosomal segments.
- Viable spermatozoa with exocytosed acrosomes: Characterized by a white (unstained) acrosomal and post-acrosomal region, indicating the occurrence of true acrosome exocytosis.
- Non-viable spermatozoa with intact acrosomes: Identified by variable staining intensities in the acrosomal region (ranging from purple to dark pink) and a characteristic dark blue or blue post-acrosomal region.
- Dead/Acrosome-reacted: These cells exhibited a clear or gray acrosomal profile alongside a blue-stained post-acrosomal segment, indicating that acrosomal loss occurred secondary to cell death (false acrosome reaction).

### Total antioxidant ability and oxidative stress analysis

To assess biochemical profiles, post-thaw semen samples (*n* = 5 per group) were centrifuged at 4,430 × *g* for 10 min at 4 °C. The resulting supernatant was carefully harvested, aliquoted, and stored at − 20 °C until analysis. Concentrations of hydrogen peroxide (H_2_O_2_), malondialdehyde (MDA), total antioxidant capacity (TAC), and catalase (CAT) were quantified using commercial diagnostic kits (Bio-Diagnostic, Giza, Egypt; Catalog Nos. HP 25, MD 2529, TA 2513, and CA 2517, respectively) on a UV–Vis spectrophotometer (UV-2602, Labomed Inc., Culver City, CA, USA) according to the manufacturer’s protocols. Absorbance values were measured at 510 nm H_2_O_2_, 534 nm (MDA), 505 nm (TAC), and 510 nm (CAT). The assays demonstrated linear ranges up to 1.5 mM/L for H_2_O_2_, 100 nmol/mL for MDA, and 2 mM/L for TAC.

### Assessment of apoptosis by flow cytometry

Apoptosis-like changes in frozen-thawed spermatozoa were evaluated using Annexin V staining and flow cytometry [40]. Sperm from each group was centrifuged to isolate the cells under cooling centrifugation system. The resulting sperm pellets were resuspended in 2 mL of binding buffer and totally mixed. A 100 µL aliquot of the sperm suspension was then mixed with 5 µL of Propidium Iodide (PI), labeled with phycoerythrin, and 5 µL of Annexin V, labeled with fluorescein isothiocyanate (FITC). The mixture was incubated in the dark for 15 min. Following incubation, 200 µL of binding buffer was added to the suspension.

To assess and quantify the apoptotic status of the spermatozoa, the stained suspensions were immediately analyzed by flow cytometry. The analysis was performed using an Accuri C6 Cytometer (BD Biosciences, San Jose, CA, USA) and its associated software equipped with a Class 1 laser emitting at a wavelength of 488 nm. A minimum of 10,000 events per sample in the target gate were acquired. FITC-labeled annexin V was detected on the FL1 channel (530/30 nm bandpass filter), while PE-labeled PI was detected in the FL2 channel (585/40 nm bandpass filter). Data acquisition and analysis were performed with Accuri C6, V1.0.264.21 software (Becton Dickinson) [41]. The proportions of positive or negative Annexin V (A-/A+), PI (PI-/PI+), and the double-positive cells were assessed. According to Peña et al. [42] spermatozoa were categorized into four classes:

- viable cells: without fluorescence signal and membrane dysfunction (A-/PI-),
- viable sperm cells with early apoptosis: viable cells labeled with Annexin V but without PI (A+/PI-),
- sperm cells with apoptosis: dead cells labeled with Annexin V and PI and with damaged permeable membranes (A+/PI+), and,
- sperm cells with necrosis: dead cells labeled with PI without Annexin V and with complete membrane loss (A-/PI+). Lastly, within each group, the spermatozoal counts for each of the specified categories were determined and documented.

### Reactive oxygen species (ROS)

The DCFDA / H2DCFDA fluorescent assay (Abcam, Cat No. ab113851) was performed to measure spermatozoa reactive oxygen species (ROS) levels according to the manufacturer’s instructions. Spermatozoa samples were washed in PBS (1 × 10^6^ cells/mL). The samples were then centrifuged at 300–500 x g for 10 min at 4 °C to remove debris. The intact cells were incubated with 10 µM H2DCFDA dye at 37 °C for 30 min in the dark to allow for cellular uptake and oxidation by intracellular ROS. After incubation, the samples were analyzed using flow cytometry on a BD FACSCanto II (BD Biosciences, San Jose, CA). Fluorescence emission was measured at 525 nm following excitation at 488 nm. Data analysis was performed using FlowJo v10 (BD Biosciences). Intact spermatozoa were gated based on scatter properties, and the population was categorized as ROS-positive or ROS-negative.

### Post-thawed microbial counts

The total bacterial count (TBC) of the semen samples was determined utilizing nutrient agar medium, according to the methodology established by [43]. Inoculated plates were incubated at 37 °C for 24 h prior to colony enumeration. *Coliform* bacteria were quantified by incubating samples at 37 °C for 16–18 h. To ensure statistical robustness, eight independent samples per group were analyzed, and the results were expressed as colony-forming units (CFU).

### Molecular docking simulations

Molecular docking simulation was performed using AutoDock 4.2 software [44]. The ligands’ 3D conformers were converted into PDBQT format by OpenBabel software, and the protein structures were prepared by adding polar hydrogens and assigning Kollman charges using AutoDock Tools. This simulation involved site-directed molecular docking, with the grid coordinates centered around the predicted active sites determined by Biovia Discovery Studio software [45]. The processes involving chlorogenic acid with mitochondrial cytochrome c (ID: Q3LUG8), HSP70 (ID: E5D621), and mitochondrial uncoupling protein 1 (ID: J9XUE4), as well as rutin with cytochrome c (ID: Q3LUG8) and mitochondrial uncoupling protein 1 (ID: J9XUE4).

For Mitochondrial cytochrome c (ID: Q3LUG8), the grid coordinates were set at (5.312, 1.801, -3.273), for HSP70 (ID: E5D621) at (-8.512, 5.914, 0.951), and for mitochondrial uncoupling protein 1 (ID: J9XUE4) at (-6.104, 0.010, 3.584), all with dimensions of 40 × 40 × 40 Å. For each ligand-protein complex, 50 docking poses were generated using the Lamarckian Genetic Algorithm, and the top-ranked conformation based on the least binding free energy (ΔG) was selected and visualized using PyMol and Biovia Discovery Studio.

### Statistical analysis

Data were initially compiled and structured in Microsoft Excel. Normal distribution and homogeneity of variance were verified using Shapiro–Wilk and Levene’s tests, respectively. Statistical processing was achieved using SPSS software (Version 25). A one-way ANOVA was then utilized to determine the significance of experimental treatments. All data are expressed as the mean ± standard error of the mean (SEM). Statistical significance was set at *P <* 0.05. When significant treatment effects were detected, Tukey’s post-hoc test was applied for multiple pairwise comparisons between treatment means.

## Results

### Effects on post-thawed sperm quality

The comparative effects of RU and CGA supplementation in a Tris-based extender on post-equilibration (5 °C for 4 h) sperm quality parameters in buffalo bulls are summarized in Table 1. The addition of RU or CGA significantly enhanced progressive motility and viability compared to the free-flavonoids treatment (*P <* 0.05). Specifically, progressive motility improved by 9.5% and 12.3%, while viability increased by 8.7% and 11.6% for the RU and CGA groups respectively, relative to the control. Sperm morphological abnormalities were not significantly affected by either treatments (*P* > 0.05). Regarding plasma membrane integrity, while 0.6 mM RU yielded a non-significant change compared to the control, CGA supplementation significantly increased membrane integrity (*P <* 0.05).

**Table 1:** Effect of phenolic compound supplementation (rutin and chlorogenic acid) in a Tris-based extender on post-equilibration (5 °C for 4 h) sperm characteristics of water buffalo (*Bubalus bubalis*) bull semen (mean ± SE, *n* = 8)

Treatment	Sperm characteristics (%)			
	Progressive motility	Viability	Membrane integrity	Abnormality
Control	73.0 ± 1.22^b^	75.8 ± 1.24^b^	74.8 ± 1.16^b^	8.6 ± 0.68
Rutin (0.6 mM)	80.0 ± 2.24^a^	82.4 ± 2.18^a^	80.6 ± 2.14^ab^	8.0 ± 0.71
Chlorogenic acid (150 µM)	82.0 ± 1.22^a^	84.6 ± 1.29^a^	82.4 ± 1.54^a^	10.2 ± 0.58
*P value*	0.01	0.01	0.02	0.09

^a-b^Means denoted with different superscripts in each column are significantly different at *P <* 0.05

### Effect on post-thaw sperm characteristics and kinematic parameters

The sperm kinematic parameters in response to the addition of RU or CGA are presented in Table 2. All supplemented groups exhibited significantly greater viability, membrane integrity, and progressive motility percentages compared to the control group (*P <* 0.05). Specifically, the DAP, DCL, and DSL were significantly higher in the CGA group compared to the control group (*P <* 0.05). In contrast, the addition of RU did not produce significant effects on these distance parameters compared to the other experimental groups (*P* > 0.05). The STR, linearity, BCF, ALH, and WOB were not significantly affected by the addition of either RU or CGA (*P* > 0.05). Regarding velocity parameters, the VAP (µm/s) and VCL (µm/s) were lowest in the RU (0.6 mM) group compared to the control group. Conversely, the VSL (µm/s) was considerably superior in the CGA group (29.5 ± 0.63) compared to the RU group (26.3 ± 0.72) (*P <* 0.005).

**Table 2:** Effects of phenolic compound supplementation (rutin and chlorogenic acid) in a Tris-based extender on post-thaw sperm characteristics and kinematic parameters in water buffalo (*Bubalus bubalis*) bull semen (mean ± SE, *n* = 8)

Item	Treatment			*P* value
	Control	Rutin (0.6 mM)	Chlorogenic acid (150 µM)	
Viability (%)	42.3 ± 0.70^b^	55.8 ± 1.40^a^	55.9 ± 1.08^a^	< 0.0001
Membrane integrity (%)	42.4 ± 0.96^b^	51.3 ± 1.24^a^	52.4 ± 1.34^a^	< 0.0001
Abnormality (%)	10.9 ± 0.91	12.0 ± 0.98	12.9 ± 0.67	0.28
PM (%)	40.3 ± 1.43^b^	51.2 ± 1.01^a^	54.6 ± 1.10^a^	< 0.0001
DAP (µm)	14.1 ± 0.68^b^	16.1 ± 0.42^a^	16.5 ± 0.34^a^	0.01
DCL (µm)	21.1 ± 1.45^b^	24.7 ± 0.85^ab^	26.0 ± 1.21^a^	0.03
DSL (µm)	10.8 ± 0.5^b^	11.5 ± 0.24^ab^	12.7 ± 0.38^a^	0.02
VAP (µm/sec)	39.7 ± 1.44^a^	34.8 ± 1.04^b^	38.6 ± 0.24^ab^	0.01
VCL (µm/sec)	63.7 ± 3.04^a^	53.2 ± 2.84^b^	60.4 ± 2.34^ab^	0.05
VSL (µm/sec)	28.4 ± 0.65^ab^	26.3 ± 0.72^b^	29.5 ± 0.63^a^	0.02
STR (%)	71.4 ± 1.69	75.0 ± 1.45	76.0 ± 1.48	0.13
LIN (%)	44.6 ± 2.34	49.4 ± 2.40	48.8 ± 2.52	0.34
WOB (%)	62.2 ± 2.06	65.2 ± 2.35	63.6 ± 2.34	0.65
ALH (µm)	2.4 ± 0.26	2.8 ± 0.18	3.0 ± 0.20	0.17
BCF (Hz)	20.3 ± 0.85	20.0 ± 0.61	22.3 ± 0.69	0.08

*PM* Progressive motility, *DCL* Distance curved line (µm), *DAP* Distance average path (µm), *DSL* Distance straight line (µm), *VCL* Velocity curved line (µm/sec), *VAP* Velocity average path (µm/sec), *VSL* Velocity straight line (µm/sec), *LIN* Linearity (VSL/VCL), *STR* Straightness (VSL/VAP), *WOB* Wobble (VAP/VCL), *BCF* Beat cross frequency (Hz) and *ALH* Amplitude of lateral head displacement (µm)

^a−b^Means denoted with different superscripts in each row are significantly different at *P <* 0.05

### Effects on acrosomal integrity

Supplementation with RU and CGA significantly increased the proportion of live sperm with intact acrosomes by 30.0% and 33.3%, respectively, compared to the control group (*P <* 0.05; Table 3). Conversely, RU and CGA treatment significantly reduced the percentages of live sperm with detached acrosomes and dead sperm with intact acrosomes relative to the control (*P <* 0.05). No significant differences were observed in the percentage of dead sperm with detached acrosomes between the treatment groups and the control (*P* > 0.05).

**Table 3:** Effect of phenolic antioxidant supplementation (rutin and chlorogenic acid) in a Tris-based extender on acrosomal integrity of post-thaw water buffalo (*Bubalus bubalis*) bull spermatozoa (mean ± SE, *n* = 5)

Treatment	Acrosomal integrity (%)			
	Live sperm with intact acrosome	Live sperm with detached acrosome	Dead sperm with intact acrosome	Dead sperm with detached acrosome
Control	40.0 ± 0.71^b^	23.8 ± 0.37^a^	31.6 ± 1.08^a^	4.6 ± 0.51
Rutin (0.6 mM)	52.0 ± 0.45^a^	21.0 ± 0.71^b^	23.2 ± 0.37^b^	3.8 ± 0.37
Chlorogenic acid (150µM)	53.2 ± 0.66^a^	21.2 ± 0.37^b^	22.2 ± 0.37^b^	3.4 ± 0.24
*P value*	< 0.0001	0.004	< 0.0001	0.13

^a−b^Means denoted within the same column with different superscripts are significantly different at *p* < 0.05

### Antioxidant status and oxidative stress biomarkers

Table 4 presents the effects of RU and CGA supplementation in a Tris-based extender on the antioxidant status and oxidative stress biomarkers of post-thaw water buffalo (*Bubalus bubalis*) bull semen. All supplemented groups exhibited significantly higher total antioxidant capacity (TAC) compared to the control groups. The peak TAC values were observed with 150 µM CGA, followed by 0.6 mM RU, relative to the control. Both treatments resulted in significantly higher catalase (CAT) levels compared to the control group (*P <* 0.01). Specifically, CAT activity increased 1.33-fold for RU and 1.67-fold for CGA relative to the control. The levels of MDA and H_2_O_2_ were significantly reduced by the inclusion of either RU or CGA compared to the control group (*P <* 0.01). Specifically, supplementation with 150 µM CGA led to a significant reduction in H_2_O_2_ compared to all other experimental groups (*P <* 0.05). Overall, CGA reduced MDA levels by 36.6% and H_2_O_2_ levels by 38.8% relative to the control group (*P <* 0.01). The RU adding reduced MDA levels by 38.9% and H_2_O_2_ levels by 19.4% relative to the control group (*P <* 0.01).

**Table 4:** Effect of phenolic compound supplementation (rutin and chlorogenic acid) in a Tris-based extender on antioxidant status and oxidative stress biomarkers in post-thaw water buffalo (*Bubalus bubalis*) bull semen (mean ± SE, *n* = 5)

Treatment	TAC (mM/L)	CAT (U/L)	MDA (nmol/mL)	H_2_O_2_ (mM/L)
Control	0.30 ± 0.020^c^	189.7 ± 11.98^b^	21.3 ± 0.53^a^	0.36 ± 0.023^a^
Rutin (0.6 mM)	0.74 ± 0.020^b^	443.7 ± 25.45^a^	13.0 ± 0.38^b^	0.29 ± 0.013^b^
Chlorogenic acid (150µM)	1.01 ± 0.043^a^	507.5 ± 14.98^a^	13.5 ± 0.24^b^	0.22 ± 0.005^c^
*P value*	< 0.0001	< 0.0001	< 0.0001	0.0001

Total antioxidant capacity (TAC), catalase (CAT), malondialdehyde (MDA), and hydrogen peroxide (H_2_O_2_)

^a−c^Means denoted within the same column with different superscripts are significantly different at *P* < 0.05

### Effects on apoptosis-like changes and intracellular ROS

**Fig. 1:**
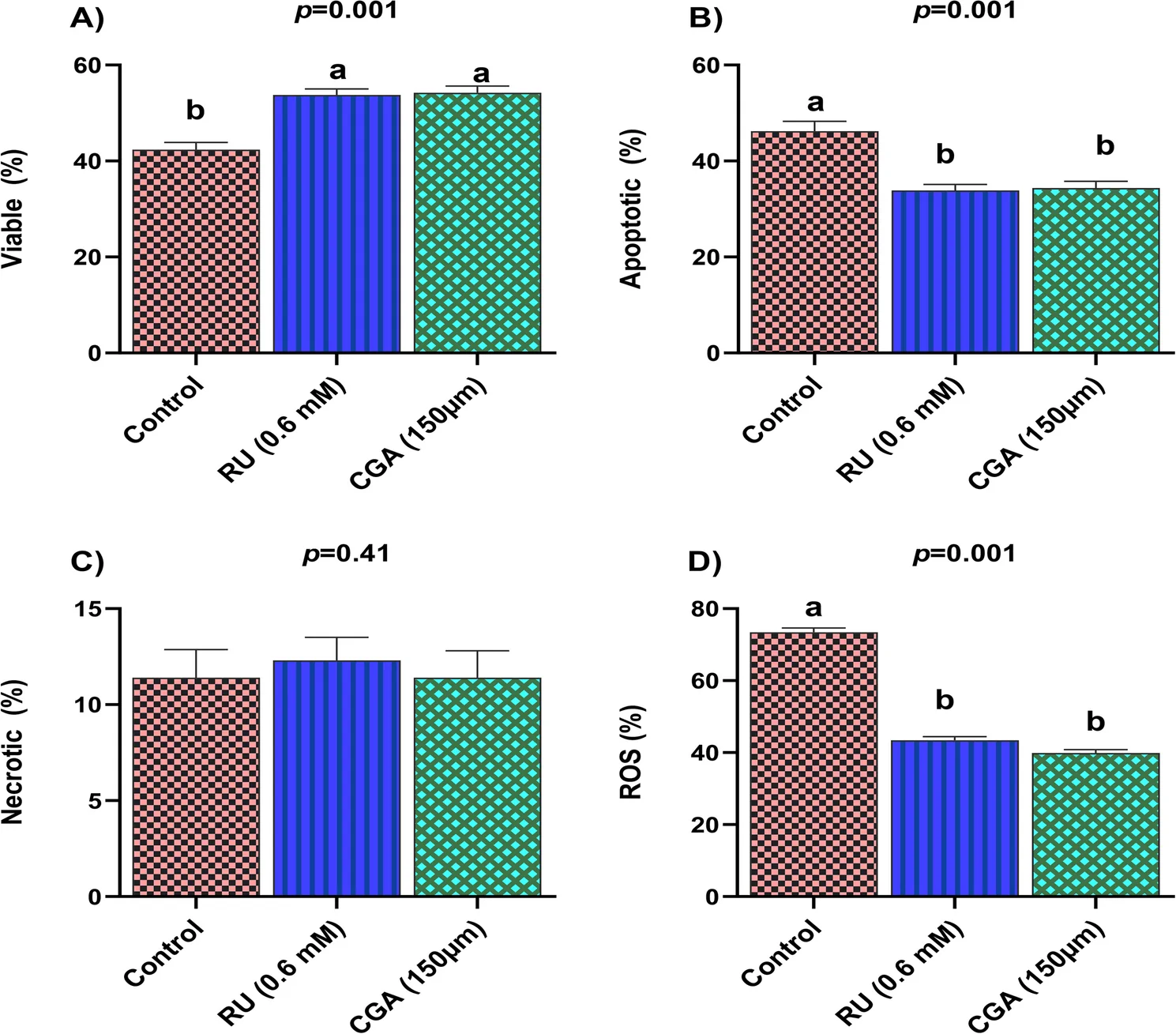
Effect of phenolic compound supplementation (rutin and chlorogenic acid) in a Tris-based extender on apoptosis-like changes (Annexin V/PI assay) and intracellular reactive oxygen species (ROS) levels in post-thaw water buffalo (*Bubalus bubalis*) bull spermatozoa (mean ± SE, *n* = 3). **A** Viable (A−/PI−), **B** Apoptotic (A+/PI+), **C** Necrotic (A−/PI+) and (**D**) ROS. ^a−b^Bars bearing different superscript letters are significantly different at *P <* 0.05

The effects of RU and CGA supplementation in a Tris-based extender on post-thaw sperm apoptosis and oxidative stress are illustrated in Fig. 1. Supplementation with either RU (26.8%) or CGA (27.8%) yielded a significantly higher percentage of viable spermatozoa compared to the control (*P <* 0.05, Fig. 1A). Furthermore, both treatments significantly reduced the proportion of apoptotic sperm (26.6% for RU and 25.5% for CGA) relative to the control group (*P <* 0.05; Fig. 1B). Necrosis percentages (Fig. 1C) showed no significant differences among the experimental groups (*P* > 0.05). Regarding oxidative status, RU and CGA supplementation significantly reduced ROS levels by 40.8% and 45.6%, respectively, compared to the control group (*P <* 0.01; Fig. 1D).

### Effects on semen microbiota

**Fig. 2:**
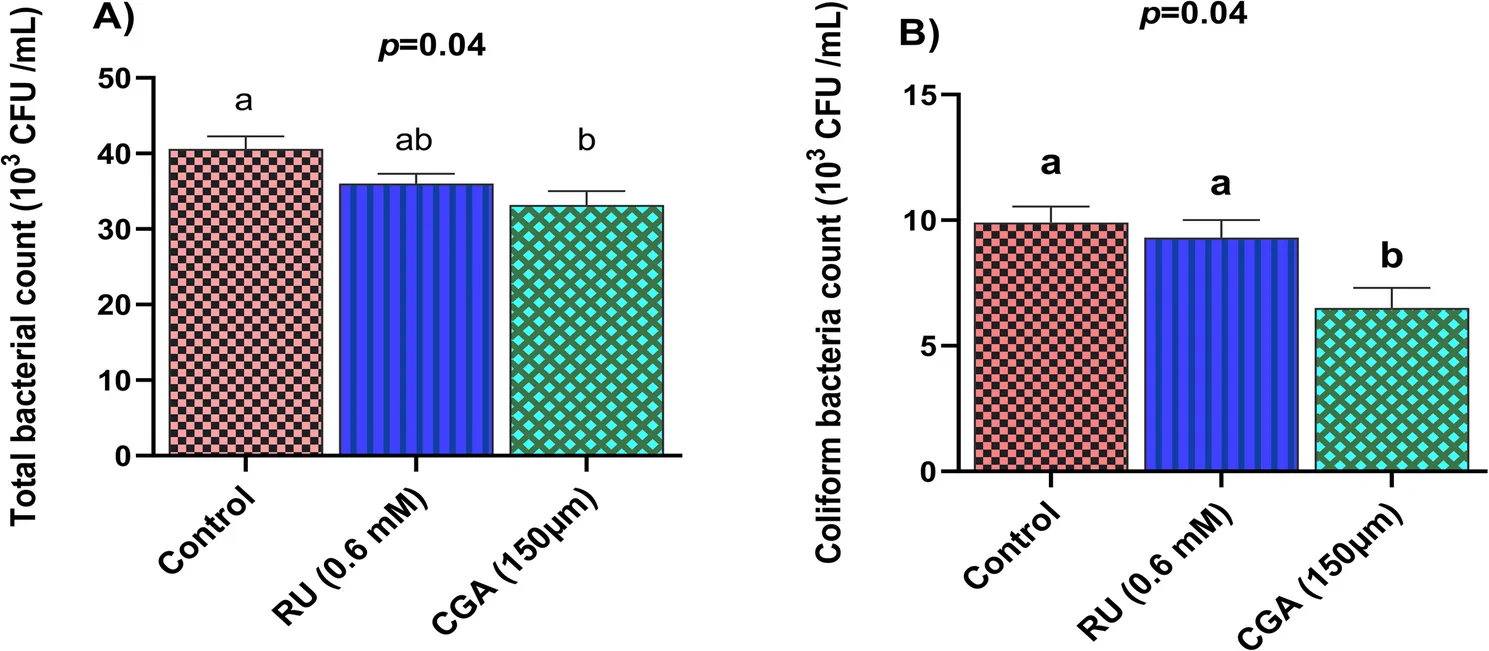
**A**-**B** Effect of phenolic compound supplementation (rutin or chlorogenic acid) in a Tris-based extender on total bacterial and (**A**) *coliform* counts (**B**) in post-thaw water buffalo (*Bubalus bubalis*) bull semen (mean ± SE, *n* = 8). ^a-b^ Bars bearing different superscript letters are significantly different at *P* <0.05

The impacts of RU and CGA on semen microbiota are presented in Fig. 2. The addition of CGA pointedly declined the total bacterial count (TBC, Fig. 2A) (33.2 *vs*. 40.6 × 10^3^ CFU/mL) compared to the control group (*P <* 0.05). While RU decreased the total bacterial count, the effect was not significant relative to the other groups. Furthermore, the *coliform* bacteria (Fig. 2B) count was significantly lower in the CGA group compared to all other experimental groups (*P <* 0.05), representing a 34.3% reduction relative to the control.

### Molecular docking results

**Fig. 3:**
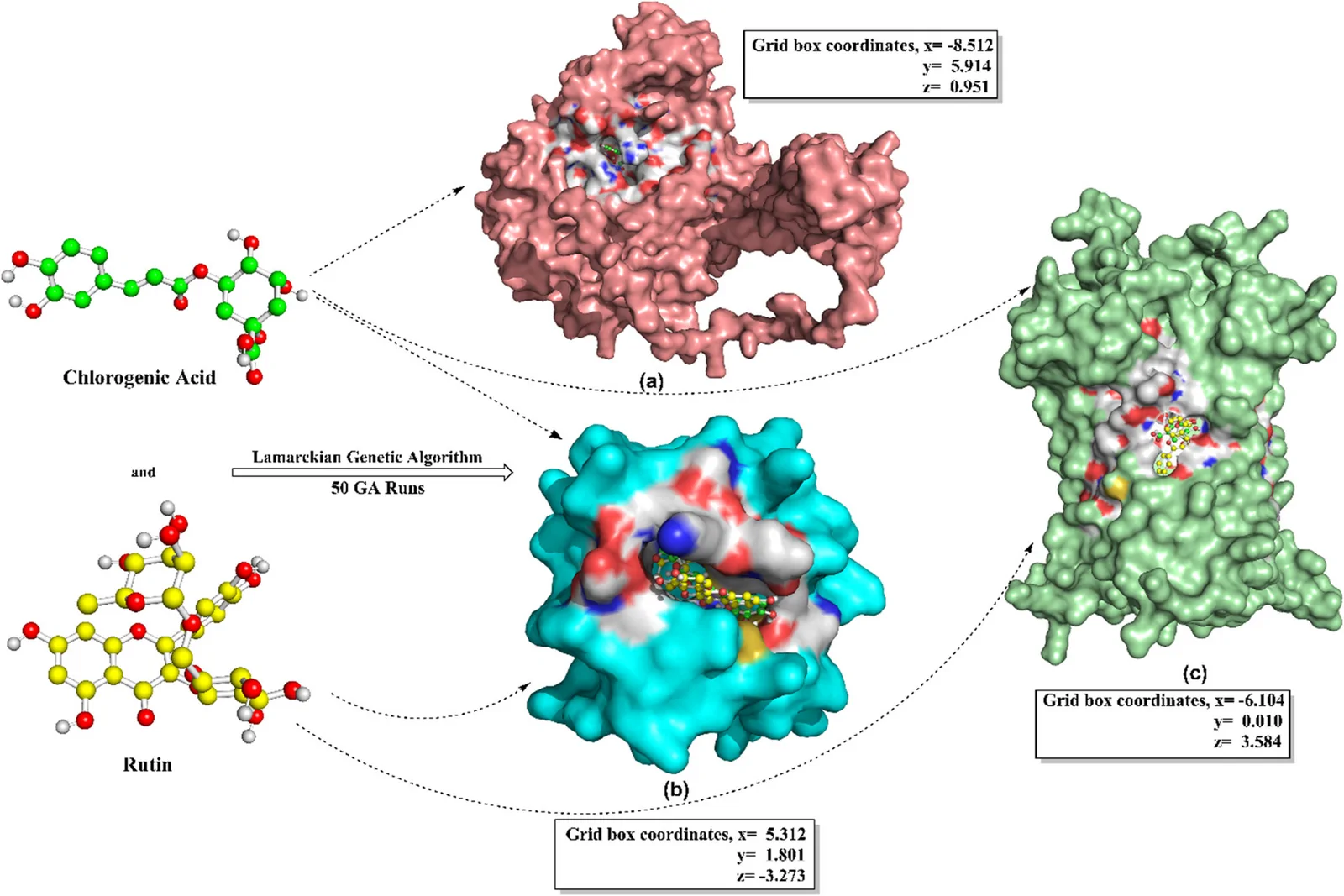
The workflow scheme that illustrates the molecular docking processes done between chlorogenic acid or rutin with (**a**) HSP70, (ID: E5D621), **b** cytochrome c (ID: Q3LUG8), and (**c**) mitochondrial uncoupling protein 1 (ID: J9XUE4)

Figure 3 illustrates the molecular docking workflow for RU and CGA interactions with heat shock protein 70 (HSP70; UniProt ID: E5D621), cytochrome c (UniProt ID: Q3LUG8), and mitochondrial uncoupling protein 1 (UCP1; UniProt ID: J9XUE4). Overall, both CGA and RU exhibited favorable binding affinities across all evaluated targets, with the strongest interactions observed against mitochondrial cytochrome c (Table 5). Specifically, CGA demonstrated a binding energy of -8.00 kcal/mol against cytochrome c, whereas RU exhibited a binding energy of -7.52 kcal/mol. Conversely, RU displayed a higher binding affinity than CGA when docked with UCP1, yielding binding energies of -5.34 kcal/mol and − 4.06 kcal/mol, respectively.

**Table 5:** The docking simulations identified the optimal binding conformers for both chlorogenic acid and rutin against the target proteins

Parameter	Mitochondrial cytochrome c (ID Q3LUG8)	
	Chlorogenic acid	Rutin
Free energy of binding (Kcal/mol).	-8.00	-7.52
Inhibition constant, Ki	1.36 µM.	3.08 µM.
Hydrogen bonding	LIGH: LYS-13(O), 1.9, 2.2 A^o^. LIGO: HIS-18(H), 3.4 A^o^. LIGO: PRO-30(H), 3.4 A^o^. LIGH: TYR-67(O), 2.9 A^o^. LIGO: THR-78(O), 2.6, 2.8, 2.9 A^o^. LIGO: THR-49(H), 2.7, 2.7, 3.3 A^o^. LIGH: SER-47(O), 3.3 A^o^. LIGH: LYS-79(O), 2.8, 3.1 A^o^.	LIGO: LYS-79(H), 3.2 A^o^. LIGH: SER-47(O), 2.5 A^o^. LIGO: ASN-52(H), 2.4, 3.4 A^o^. LIGO: HIS-18(H), 2.9, 3.0 A^o^. LIGO: LYS-79(H), 2.6, 3.2, 3.3 A^o^. LIGH: LYS-81(O), 1.9 A^o^.
Chlorogenic acid with HSP70 (ID: E5D621)		
Free energy of binding (Kcal/mol).	-4.71	
Inhibition constant, Ki	353.46 µM	
Hydrogen bonding	LIGO: SER-275(H), 1.9 A^o^. LIGO: SER-340(H), 2.6 A^o^. LIGO: THR-14(H), 2.4 A^o^. LIGO: THR-15(H), 2.4 A^o^. LIGH: ASP-234(O), 2.1 A^o^. LIGO: LYS-271(H), 1.9, 2.7 A^o^.	

**Parameter**	**Mitochondrial uncoupling protein 1 (ID: J9XUE4)**	
	**Chlorogenic acid**	**Rutin**
Free energy of binding (Kcal/mol).	-4.06	-5.34
Inhibition constant, Ki	1.05 mM	122.57 µM
Hydrogen bonding	LIGO: ARG-96(H), 2.0 A^o^. LIGO: ARG-88(H), 1.9 A^o^. LIGO: ARG-279(H), 2.0, 2.3 A^o^. LIGH: ASP-28(O), 1.9, 2.5 A^o^.	LIGH: GLU-193(O), 2.1, 2.6 A^o^. LIGH: GLN-89(O), 2.3 A^o^. LIGO: ARG-88(H), 2.2, 2.7, 2.8 A^o^. LIGO: LYS-239(H), 2.3 A^o^. LIGH: ASN-182(O), 2.3 A^o^. LIGH: ASP-28(O), 2.5 A^o^. LIGO: ARG-279(H), 2.1, 2.3 A^o^. LIGO: LYS-140(H), 1.7 A^o^.

**Fig. 4:**
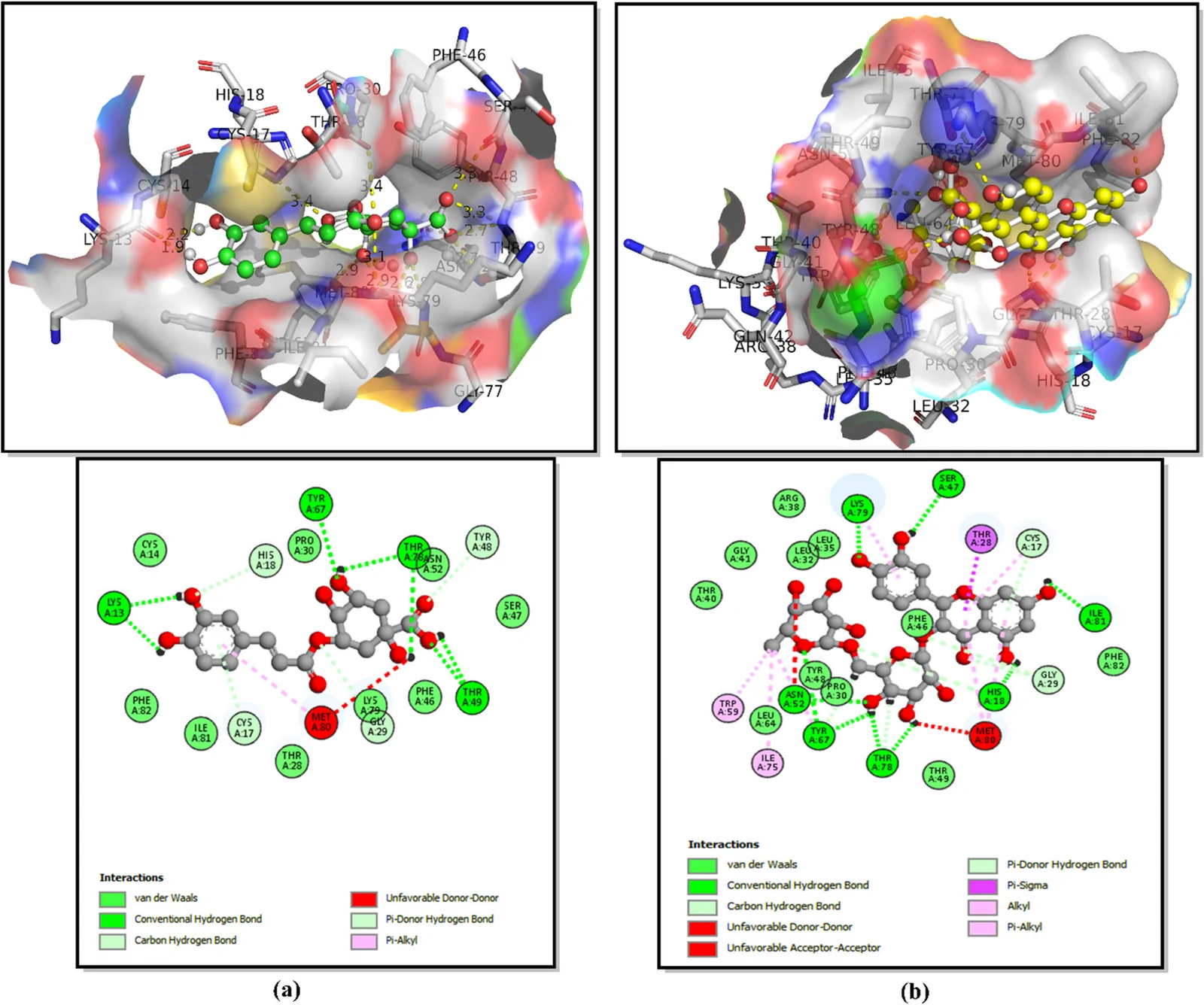
The 3D poses and the 2D interaction maps of the best conformers of chlorogenic acid (**a**) and rutin (**b**) with mitochondrial uncoupling protein 1(ID: J9XUE4 protein)

**Fig. 5:**
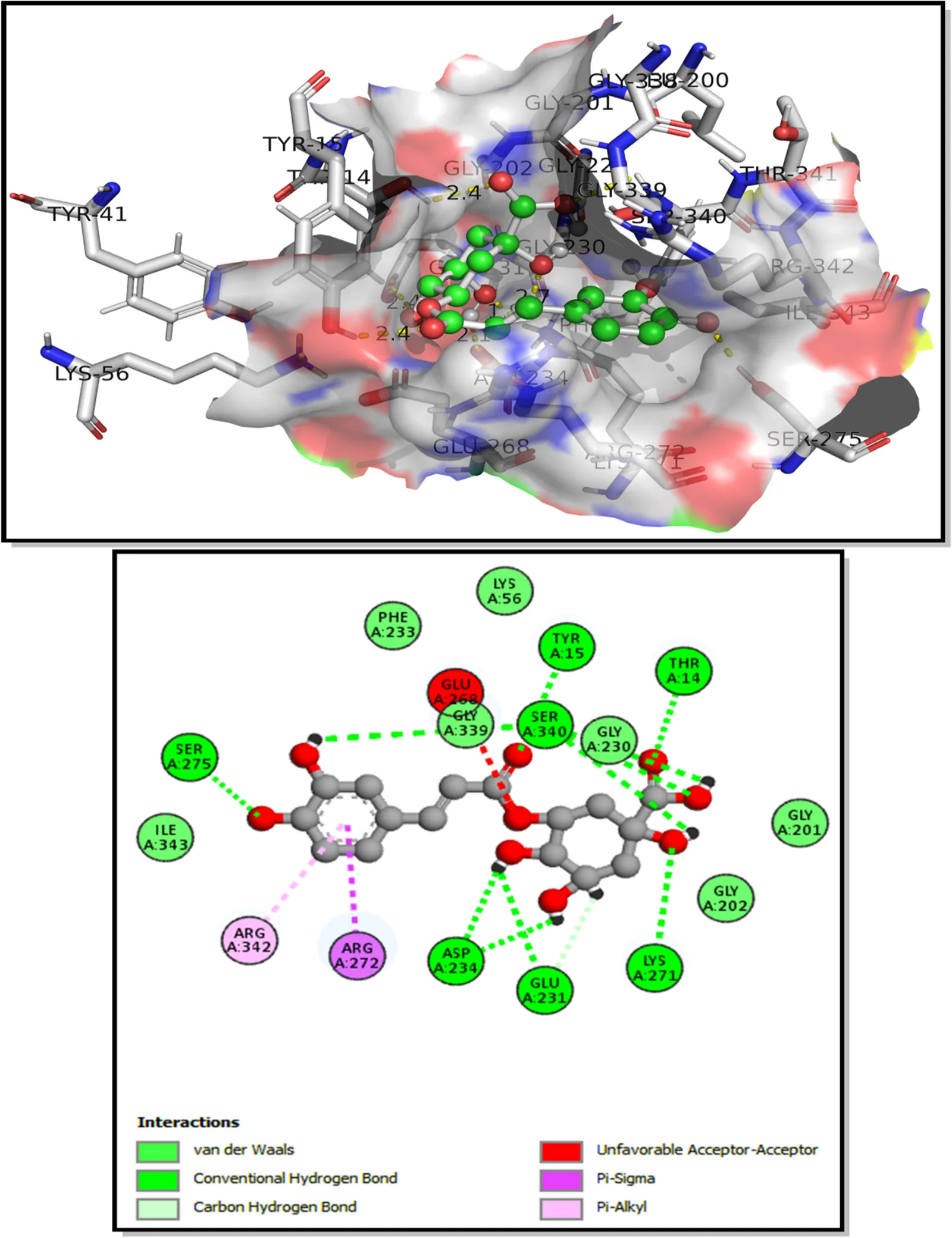
The 3D visual pose and 2D interaction network of the top-ranked chlorogenic acid conformer docked with HSP70 (ID: E5D621)

**Fig. 6:**
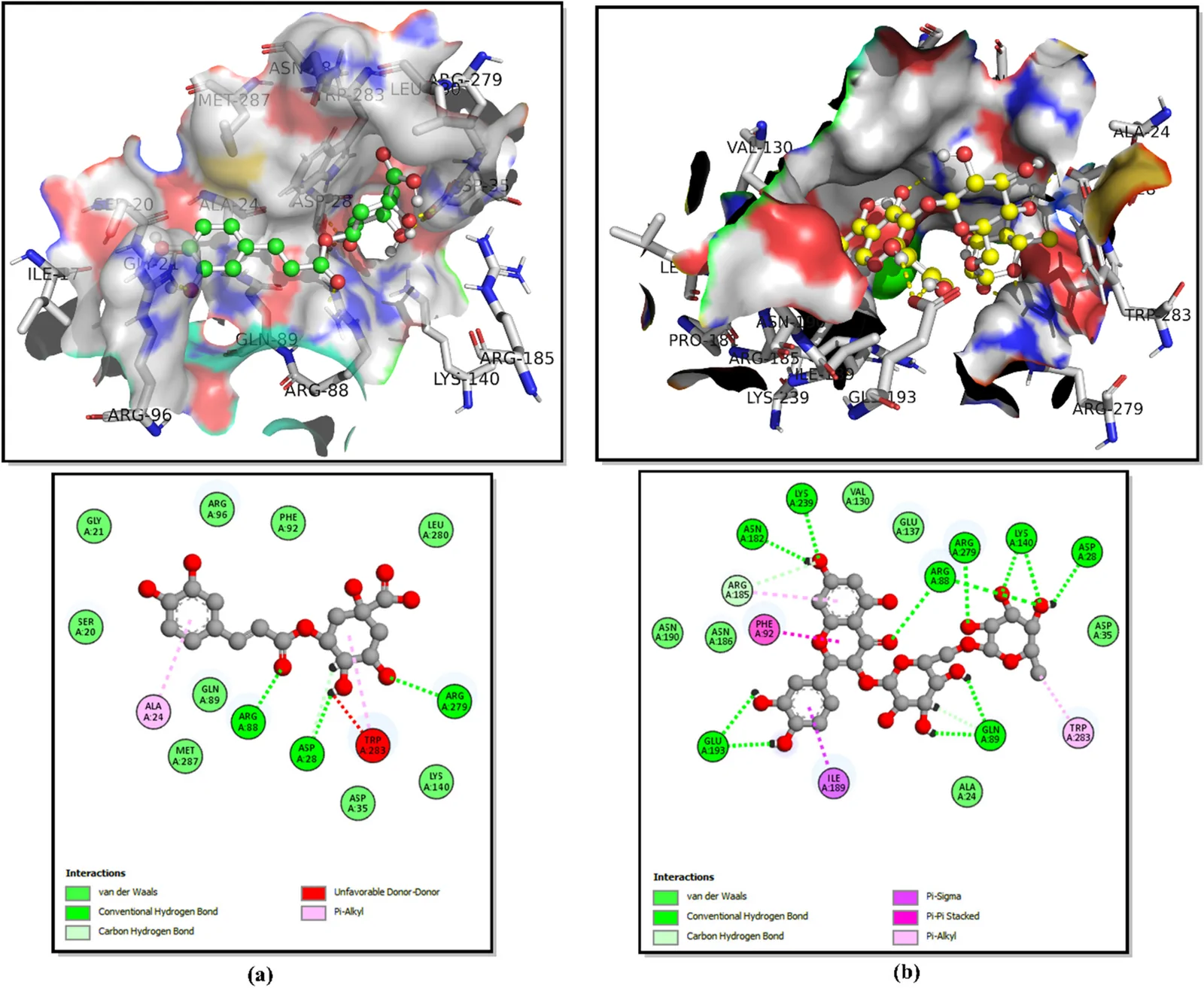
Molecular docking analysis of target compounds with mitochondrial cytochrome *c* (UniProt ID: Q3LUG8). The 3D binding orientations and corresponding 2D ligand-protein interaction networks are displayed for chlorogenic acid (**a**) and rutin (**b**)

The 3D binding poses and corresponding 2D ligand–protein interaction networks of CGA (Fig. 4a) and RU (Fig. 4b) with UCP1 are depicted in Fig. 4. Among non-covalent forces including van der Waals forces and electrostatic attractions hydrogen bonding served as the primary stabilizing interaction, with specific contact frequencies detailed in Table 5. Furthermore, the 3D binding orientation and 2D interaction network for the top-ranked CGA conformer docked with HSP70 are shown in Fig. 5. Finally, Fig. 6 displays the 3D visual poses and 2D interaction maps of the best-scoring conformers of CGA (Fig. 6a) and RU (Fig. 6b) complexed with mitochondrial cytochrome c. This predicted binding profile provides a plausible hypothesis for the observed antioxidant effects.

## Discussion

Cryopreservation is a pivotal reproductive biotechnology for enhancing reproductive efficiency and genetic progress in livestock. While this process offers major advantages such as accelerating genetic improvement and overcoming geographical barriers it frequently induces detrimental changes in sperm quality post-thawing [4]. This decline is primarily due to the overproduction of ROS, leading to oxidative stress. Such stress induces sperm dysfunction, including damage to the plasma membrane and acrosome, which ultimately results in DNA fragmentation and reduced fertilizing capacity [14, 22, 38]. To optimize the cryopreservation process, various exogenous antioxidants are integrated into freezing extenders to neutralize ROS, boost endogenous antioxidant defense systems, and preserve sperm fertility. Among these, plant-derived flavonoids the primary bioactive polyphenols in plants exhibit remarkable therapeutic potential owing to their potent antioxidant, anti-inflammatory, and anti-apoptotic properties.

In this study, we evaluated the comparative antioxidant, anti-apoptotic, and antimicrobial effects of RU and CGA on the cryotolerance of buffalo spermatozoa. Supplementation with either compound significantly enhanced post-thaw progressive motility, viability, and plasma membrane integrity. The cryoprotective efficacy of these additives was further evidenced by the preservation of acrosomal integrity and a significant reduction in apoptotic cell populations. Crucially, these improvements were mediated by a mitigation of oxidative stress evidenced by reduced ROS, MDA, and H_2_O_2_ alongside an improvement in endogenous antioxidant defense system via elevated TAC and catalase activity. Notably, CGA exhibited potent antibacterial properties, significantly reducing total bacterial counts and *coliform* levels, thereby enhancing the hygienic quality of extended semen.

In silico docking analyses predictions suggest a potential interactions between both polyphenols and key target proteins regulating sperm mitochondrial bioenergetics and antioxidant response pathways. Specifically, CGA revealed a stronger prediction binding affinity toward mitochondrial cytochrome c than RU, yielding binding energies of -8.00 and − 7.52 kcal/mol, respectively. Conversely, RU propose a higher binding affinity than CGA when docked against UCP1, exhibiting a more prediction favorable interaction binding energy (-5.34 *vs.* -4.06 kcal/mol) and a lower inhibition constant (Ki) compared to CGA. Finally, CGA complexed with HSP70 with a binding energy of -4.71 kcal/mol.

Assessment of progressive motility, viability, and plasma membrane integrity provides a comprehensive profile of spermatozoal functional competence, serving as a critical predictor of fertilizing capacity. The present study demonstrates that supplementation with either RU or CGA significantly enhances sperm motility, viability, and plasma membrane integrity, while concurrently slight improving in kinematic parameters relative to the control group. Because motility is intrinsically dependent on robust mitochondrial bioenergetics, these improvements likely reflect preserved mitochondrial functionality. Consistent with our observations, RU supplementation (0.4–0.6 mM) has been reported to significantly improve sperm motility, mitochondrial activity, and both membrane and acrosomal integrity in post-thaw boar spermatozoa [30]. Furthermore, previous work evaluating buffalo semen cryopreservation demonstrated that RU or CGA inclusion in freezing extenders significantly improved total and progressive motility, kinematic traits, viability, and plasma membrane functionality while mitigating DNA damage [12], which aligns closely with our present findings.

Rutin has been shown to downregulate the expression of dynamin-related protein 1 (Drp1), a key mediator of mitochondrial fission [46]. Mitochondrial dynamics are essential for maintaining the metabolic demands of the cell; specifically, there is a strong positive correlation between progressive motility and mitochondrial efficiency, as the latter drives the production of ATP required for flagellar movement [47]. While mitochondrial health is vital, some research indicates that excessive or improper Drp1 inhibition can lead to mitochondrial dysfunction, ultimately impairing the sperm’s capacity for motility and successful fertilization [48]. The lower VAP and VCL observed with RU treatment suggest a physiological trade-off between its antioxidant protection and sperm kinematics [12]. While RU maintains membrane integrity and controls oxidative stress [31], potent radical scavenging may suppress reactive oxygen species ROS below levels required for hyperactivation signaling [49]. High flavonoid concentrations can also increase medium viscosity, cause mechanical drag, or impair calcium-dependent flagellar motility [50, 51]. Additionally, RU binding may reduce lipid bilayer fluidity, resulting in structural rigidification that constrains lateral head movement [50]. Crucially, these attenuated velocities reflect a stabilized, energy-conserving state that protects against premature hyperactivation and subsequent exhaustion, ultimately prolonging sperm viability [52].

In the present study, the improvement in sperm motility observed with RU supplementation is largely attributable to its potent antioxidant activity, which neutralizes oxidative stress and mitigates cryo-damage. These findings align with previous studies demonstrating that RU alleviates sulfasalazine-induced male infertility by enhancing gonadotropin secretion and improving sperm motility [28]. Furthermore, RU protects against mercuric chloride-induced reproductive toxicity in male rats by upregulating endogenous antioxidant defenses, attenuating oxidative stress, and reducing sperm necrosis [53]. Similarly, CGA supplementation has been reported to enhance progressive motility while reducing DNA damage and oxidative stress markers in preserved human semen [33], an effect largely driven by Nrf2 signaling pathway activation. Likewise, supplementing ram semen extenders with 0.8 mg/mL CGA significantly improved progressive motility, plasma membrane integrity, and acrosomal status [34]. Mechanistically, CGA enhances progressive motility by preserving mitochondrial ATP generation, thereby providing the metabolic energy required for forward flagellar propulsion.

Oxidative stress remains a primary challenge compromising sperm quality during the cryopreservation process. The present study demonstrates that the supplementation of either RU or CGA markedly reduced the levels of ROS, MDA, and H_2_O_2_, while simultaneously enhancing the TAC and CAT activity in cryopreserved buffalo semen. Similarly, the addition of RU (up to 1.0 mM) significantly attenuated ROS accumulation and MDA production by strengthen the activity of antioxidant enzymes, including SOD, CAT, and GSH-Px, in cryopreserved boar spermatozoa [30]. Likewise, fortifying buffalo freezing extenders with either RU (0.4 mM) or CGA (150µM) was shown to significantly diminish MDA levels while restoring SOD, CAT, and GPx activities, alongside elevating reduced glutathione (GSH) and TAC concentrations [12].

The potent antioxidant efficacy of RU (quercetin-3-O-rutinoside) is fundamentally anchored in its chemical structure as a flavonoid glycoside [54]. Rich in phenolic hydroxyl groups, this structure enables a multi-faceted cytoprotective defense through direct free-radical scavenging, pro-oxidant metal ion chelation, and upregulation of endogenous antioxidant pathways [54]. By binding transition metals, RU prevents metal ion-induced lipid peroxidation. Furthermore, RU-mediated mitigation of oxidative stress and restoration of CAT and intracellular GSH levels have been validated in rat models of cisplatin-induced reproductive toxicity [25, 26]. At the molecular level, these antioxidant-potentiating effects are largely driven by activation of the Nrf2/ARE signaling cascade, which triggers the transcriptional expression of downstream cytoprotective enzymes [29]. Finally, the marked attenuation of ROS observed in the present study may also involve RU’s capacity to inhibit xanthine oxidase a key enzymatic source of superoxide radical generation [55].

Chlorogenic acid (CGA) is a prominent phenolic compound recognized for its diverse health-promoting properties in both human and animal physiology [32, 56]. These therapeutic benefits stem primarily from its potent antioxidant, anti-inflammatory, anti-apoptotic, and cytoprotective activities [32]. By mitigating oxidative stress and preserving mitochondrial integrity, CGA optimizes spermatozoal bioenergetics, thereby enhancing overall motility and kinetic performance. These observations align with previous findings where CGA supplementation significantly increased total motility in boar spermatozoa during extended liquid storage at 17 °C for up to 72 h [57]. Similarly, nano-encapsulated antioxidants such as alpha-lipoic acid have been shown to enhance buffalo spermatozoal cryotolerance by strengthening antioxidant status and preserving mitochondrial function [18]. Furthermore, supplementing ram semen extenders with CGA markedly suppressed ROS accumulation while upregulating key antioxidant parameters, including CAT, SOD, and TAC [34]. Structurally, CGA contains five hydroxyl groups and a carboxyl group [35]; its phenolic hydroxyl moieties exhibit high reactivity toward free radicals, which underpins its exceptional radical-scavenging capacity.

Cryopreservation-induced programmed cell death is mediated by a series of biochemical shifts, including the triggering of the caspase cascade and mitochondrial dysfunction. Monitoring these transitions through flow cytometric analysis is essential for refining cryoprotective strategies. In the present study, supplementing buffalo freezing extenders with either RU or CGA significantly increased sperm viability while reducing the proportion of apoptotic spermatozoa, demonstrating potent anti-apoptotic efficacy. These findings align with earlier reports where flavonoid supplementation, such as chrysin, enhanced viability and attenuated apoptosis in cryopreserved rabbit spermatozoa [22]. Furthermore, the anti-apoptotic action of RU is well-documented to occur via the inhibition of caspase-3 and caspase-9 activities alongside the upregulation of the anti-apoptotic protein B-cell lymphoma 2 (Bcl-2) [29]. Our findings demonstrate that CGA exerts a potent anti-apoptotic effect on cryopreserved buffalo sperm. This is evidenced by a significant increase in sperm viability and a reduction in necrotic rates. Mechanistically, CGA mitigates mitochondrial damage, promotes BCL-2 expression, and suppresses both Bax upregulation and cytochrome c release [13, 58]. This prevents caspase activation and inhibits the expression of caspase-independent pathway components.

RU is hydrolyzed by the gut microbiota into its active aglycone, quercetin, and the disaccharide rutinose [29]. Regarding its antimicrobial action, CGA exerts potent bactericidal activity by directly binding to bacterial cell membranes and disrupting their structural integrity. This membrane destabilization leads to the leakage of essential intracellular metabolites, dissipation of the trans-membrane potential, and eventual cell lysis [59]. Furthermore, CGA impairs bacterial viability by targeting the shikimate pathway and disrupting bioenergetic metabolism through the dual inhibition of the tricarboxylic acid cycle and oxidative ATP synthesis [34]. Additionally, RU failed to reduce the bacterial load in post-thawed buffalo semen. The inability of RU to reduce bacterial load may stem from its high molecular weight, low aqueous solubility, and specific chemical structure, which collectively prevent it from penetrating bacterial cell walls.

To gain a deeper understanding of how these molecules interact with key functional proteins in buffalo sperm, an in silico simulation study was conducted. The molecular docking analysis suggested strong docking predictions of CGA and RU toward proteins regulating sperm antioxidant status and mitochondrial function. Specifically, CGA and RU presented favorable predictions interactions with mitochondrial cytochrome c and uncoupling protein 1 (UCP1), respectively, while CGA also suggest a binding energy of -4.71 kcal/mol with heat shock protein 70 (HSP70). Beyond its classical role in thermogenesis, UCP1 functions as a vital metabolic modulator in spermatozoa, critically governing cellular bioenergetics and sperm motility by optimizing the mitochondrial proton gradient and ATP synthesis [60]. Conversely, cryopreservation can trigger mitochondrial-mediated apoptosis, characterized by the accelerated release of cytochrome c into the cytosol, which subsequently activates the intrinsic apoptotic pathway [61, 62]. These docking predictions suggest a potential molecular basis for the observed antioxidant activity. Overall, these in silico findings corroborate our in vitro experimental observations. While these molecular docking simulations provide valuable structural insights into potential ligand-receptor interactions, we acknowledge that computational modeling alone cannot definitively establish biological mechanisms. In the absence of direct functional assays (such as enzyme inhibition or receptor binding kinetics), these findings should be interpreted as hypothesis-generating predictions. Future functional and mechanistic experiments are required to validate these predicted binding modes in biological systems.

This study has certain limitations, particularly regarding the investigation of potential genetic alterations resulting from the fortification of freezing media with these antioxidants. According to the results the CGA has a stronger binding affinity for mitochondrial cytochrome c, whereas rutin binds more effectively to UCP1. Additionally, CGA demonstrated potent antibacterial activity, while RU underperformed relative to the control in certain kinematic parameters (e.g., VAP, VCL). These complementary mechanisms suggest that a dual CGA–RU treatment may yield synergistic or additive benefits for frozen semen quality, highlighting a promising avenue for further experimental validation. Furthermore, due to resource constraints, we were unable to conduct in vivo fertility assays to validate the laboratory findings. These areas represent important directions for future research.

## Conclusion

The results suggest that fortifying buffalo semen freezing media with antioxidant flavonoids (rutin or chlorogenic acid) markedly enhances sperm motility, acrosomal integrity, and plasma membrane functionality. These improvements were accompanied by a significant reduction in apoptotic spermatozoa, oxidative stress markers (MDA, and H_2_O_2_) and ROS levels. Additionally, the supplementation of these flavonoids significantly enhanced overall antioxidant status, as evidenced by increased activity of CAT and levels of TAC. Adding chlorogenic acid significantly decreased total bacterial and *coliform* counts relative to the control extender. Molecular docking predictions suggest binding affinities of chlorogenic acid and rutin toward key target proteins governing spermatozoal antioxidant status and mitochondrial function. While these in silico findings highlight the potential of both polyphenols to optimize semen cryopreservation extenders, further research is warranted to validate their in vivo reproductive efficacy and elucidate their precise molecular mechanisms through multi-omics approaches.

## Data Availability

All data included in this study are presented in the form of tables and figures.

## Abbreviations

CGA: Chlorogenic acid
RU: Rutin
ARTs: Assisted reproductive technologies
IVM: In vitro oocyte maturation
IVEP: In vitro embryo production
OS: Oxidative stress
PUFA: Polyunsaturated fatty acid
SPM: Sperm progressive motility
HOST: Hypo-osmotic swelling test
CASA: Computer-Assisted Sperm Analysis
DSL: Distance straight line
LIN: Linearity
DCL: Distance curved line
VAP: Velocity average path
DAP: Distance average path
STR: Straightness
VSL: Velocity straight line
WOB: Wobble
BCF: Beat cross frequency
ALH: Amplitude of lateral head displacement
H_2_O_2_: Hydrogen peroxide
MDA: Malondialdehyde
TAC: Total antioxidant capacity
CAT: Catalase
ROS: Reactive oxygen species
TBC: Total bacterial count
CFU: Colony-forming units
HSP70: Heat shock protein 70
Drp1: Dynamin-related protein 1
GSH: Reduced glutathione
Bcl-2: B-cell lymphoma 2
UCP1: Uncoupling protein 1
